# CCN2 Activates Cellular Senescence Leading to Kidney Fibrosis in Folic Acid-Induced Experimental Nephropathy

**DOI:** 10.3390/ijms26094401

**Published:** 2025-05-06

**Authors:** Lucia Tejedor-Santamaria, Laura Marquez-Exposito, Alicia Villacampa, Vanessa Marchant, Antonio Battaglia-Vieni, Sandra Rayego-Mateos, Raul R. Rodrigues-Diez, Fatima Milhano Santos, Floris A. Valentijn, Sebastian N. Knoppert, Roel Broekhuizen, María Piedad Ruiz-Torres, Roel Goldschmeding, Alberto Ortiz, Concepción Peiró, Tri Q. Nguyen, Adrián M. Ramos, Marta Ruiz-Ortega

**Affiliations:** 1 Molecular and Cellular Biology in Renal and Vascular Pathology, Department of Medicine, IIS-Fundación Jiménez Díaz, Universidad Autónoma Madrid, 28040 Madrid, Spain; lucia.tejedor@quironsalud.es (L.T.-S.); laura.mar.exp@gmail.com (L.M.-E.); vanessa.marchant@uam.es (V.M.); antonio.battaglia@uam.es (A.B.-V.); srayego@quironsalud.es (S.R.-M.); fatima.milhano@quironsalud.es (F.M.S.); 2 Instituto de Salud Carlos III., 28029 Madrid, Spain; aortiz@fjd.es (A.O.); amramos@fjd.es (A.M.R.); 3 Department of Pharmacology, School of Medicine, Universidad Autónoma de Madrid, 28029 Madrid, Spain; alicia.villacampa@idipaz.es (A.V.); concha.peiro@uam.es (C.P.); 4 Vascular Pharmacology and Metabolism (FARMAVASM) Group, IdiPAZ, 28029 Madrid, Spain; 5 Department of Cell Biology, School of Medicine, Complutense University, 28040 Madrid, Spain; raulro11@ucm.es; 6 Department of Pathology, University Medical Center Utrecht, H04.312, Heidelberglaan 100, 3584 CX Utrecht, The Netherlands; f.a.valentijn@gmail.com (F.A.V.); s.n.knoppert-3@umcutrecht.nl (S.N.K.); r.broekhuizen.umcu@gmail.com (R.B.); r.goldschmeding@umcutrecht.nl (R.G.); t.q.nguyen@umcutrecht.nl (T.Q.N.); 7 Department of Systems Biology, University of Alcalá, Alcalá de Henares, 28871 Madrid, Spain; mpiedad.ruiz@uah.es; 8 Division of Nephrology and Hypertension, IIS-Fundación Jiménez Díaz, Universidad Autónoma Madrid, 28049 Madrid, Spain

**Keywords:** CCN2, senescence, fibrosis, CKD, AKI, microvascular rarefaction

## Abstract

Cellular communication network factor 2 (CCN2, also known as CTGF) is a complex protein that regulates numerous cellular functions. This biomolecule exhibits dual functions, depending on the context, and can act as a matricellular protein or as a growth factor. CCN2 is an established marker of fibrosis and a well-known mediator of kidney damage, involved in the regulation of inflammation, extracellular matrix remodeling, cell death, and activation of tubular epithelial cell (TECs) senescence. In response to kidney damage, cellular senescence mechanisms are activated, linked to regeneration failure and progression to fibrosis. Our preclinical studies using a total conditional CCN2 knockout mouse demonstrate that CCN2 plays a significant role in the development of a senescence phenotype after exposure to a nephrotoxic agent. CCN2 induces cell growth arrest in TECs, both in the early phase and in the chronic phase of folic acid nephropathy (FAN), associated with cell-death/necroinflammation and fibrosis, respectively. Renal CCN2 overexpression was found to be linked to excessive collagen accumulation in tubulointerstitial areas, microvascular rarefaction, and a decline in renal function, which were observed three weeks following the initial injury. All these findings were markedly diminished in conditional CCN2 knockout mice. In the FAN model, injured senescent TECs are associated with microvascular rarefaction, and both were modulated by CCN2. In primary cultured endothelial cells, as previously described in TECs, CCN2 directly induced senescence. The findings collectively demonstrate the complexity of CCN2, highlight the pivotal role of cellular senescence as an important mechanism in renal injury, and underscore the critical function of this biomolecule in kidney damage progression.

## 1. Introduction

Chronic kidney disease (CKD) is a common, progressive, and irreversible disease that has been estimated to become the 5th global cause of death by 2040 [1]. The current strategies for CKD patients include pharmacological interventions (SGLT2 or renin-angiotensin system inhibitors) as well as lifestyle changes (exercise, diet, and others). However, these strategies only slow the progression of the disease, and, therefore, better therapeutic options are still needed [2]. Moreover, acute kidney injury (AKI) has no effective treatment, and following an AKI episode, many patients progress to CKD [1,2,3,4]. Hence, basic and preclinical research is needed to understand the AKI-to-CKD transition to develop new therapeutic strategies that prevent the disease progression or even restore the renal function.

A senescent cell is stuck in a unique state, not properly functional, characterized by prolonged cell cycle arrest (CCA) that prevents cells from entering apoptosis [5,6]. These cells produce a specific secretome, so-called senescence-associated secretory phenotype or “SASP”, containing a large range of cytokines, chemokines, and matrix-degrading proteases that could contribute to secondary senescence and damage amplification, although SASP-specific composition varies based on cell type and pathological condition [7,8,9]. The senescence process can be induced in different ways: repetition of cell division and telomere shortening, oxidative stress, or genotoxic damage [10,11], as well as by cytokines and growth factors [6,12]. Cellular senescence is a mechanism activated in different biological processes, such as tissue regeneration and repair, embryonic development, protection against cancer, and fibrosis [5]. Regarding the kidney, an increase in the number of senescent cells has been observed in AKI and CKD and in aging kidneys [6]; therefore, strategies targeting senescence have been proposed to prevent kidney damage.

Cellular communication network factor 2 (CCN2) was previously known as connective tissue growth factor (CTGF). However, as a member of the CCN matricellular protein family, it shares structural and biological functions, and thus its name has officially changed [13]. CCN2 is a key extracellular matrix (ECM) component and, as a matricellular protein, participates in ECM homeostasis and cellular communication [4,14]. Besides that, CCN2 is a growth factor or cytokine, eliciting pleiotropic effects, such as ECM synthesis, angiogenesis, proinflammatory responses, and cell growth regulation, including cellular senescence [15,16,17,18,19].

Several groups have investigated the molecular mechanisms elicited by CCN2 and its role in different diseases. Regarding the kidney, CCN2 could modulate proinflammatory, profibrotic, or cell-death pathways and therefore contribute to renal damage progression [16,19,20,21,22,23]. Many preclinical studies targeting CCN2, by neutralizing antibodies, antisense oligonucleotides, gene silencing, or studies in knockout mice, have proposed CCN2 as a therapeutic target in experimental kidney damage [24,25,26]. CCN2 is also involved in senescence-associated processes. This protein is not only a prominent SASP factor secreted in large amounts by senescent cells, but it can also act by itself as an inducer of cellular senescence in vitro [27,28]. Importantly, in primary cultured tubular epithelial cells (TECs), we have described that CCN2 directly induced tubular cell senescence, as shown by direct stimulation with CCN2 recombinant protein as well as *Ccn2* gene silencing in hypoxic cells [19]. After an AKI episode, the impairment recovery of injured TECs can result in a senescent phenotype change, characterized by CCA and increased release of SASP components, in particular proinflammatory and profibrotic factors, that contribute to the AKI-to-CKD progression [2]. In human kidney transplant biopsies, there is an association between renal function decline, CCN2 expression, and senescence markers in TECs [19]. Moreover, CCN2 can activate the nuclear factor-κB (nuclear factor-κB) pathway in the murine kidney [16], a key molecular mechanism driving the overexpression of SASP-related genes. In the model of ischemia-reperfusion injury (IRI) in CCN2-deficient mice, we have described the role of this protein in the activation of cellular senescence associated with redox/NRF2 pathway regulation in the AKI phase and linked it to fibrosis in the chronic phase of the disease [19,29]. CCN2 can bind to TECs in vivo and activate downstream signaling [30]. Furthermore, studies employing a specific conditional deletion of CCN2 in tubular cells have shown a reduction in renal fibrosis in experimental IRI [31], remarking that TECs are important target cells of CCN2 actions in the kidney. Some evidence suggests that injured TECs can also promote pericyte detachment, loss of nearby capillaries, and microvascular rarefaction [32,33]. CKD patients present endothelial dysfunction and accelerate vascular aging [34]. In an early study, we described that CCN2 can induce endothelial dysfunction in mouse aorta [35]. However, the involvement of CCN2 in modulating microvascular rarefaction or inducing senescence in endothelial cells has not been investigated. Therefore, to gain insight into the mechanism involved in the AKI-to-CKD transition, we have investigated senescence-related mechanisms, regeneration failure, microvascular rarefaction, and progression to kidney fibrosis in a model of folic acid-induced nephrotoxicity (FAN) in tamoxifen-inducible CCN2 full knockout mice as well as the direct effect of CCN2 on endothelial senescence by in vitro studies in primary cultures of human endothelial cells. 

## 2. Results

### 2.1. CCN2 Deficiency Diminishes Kidney Damage in Chronic Folic Acid Nephropathy

**Figure 1:**
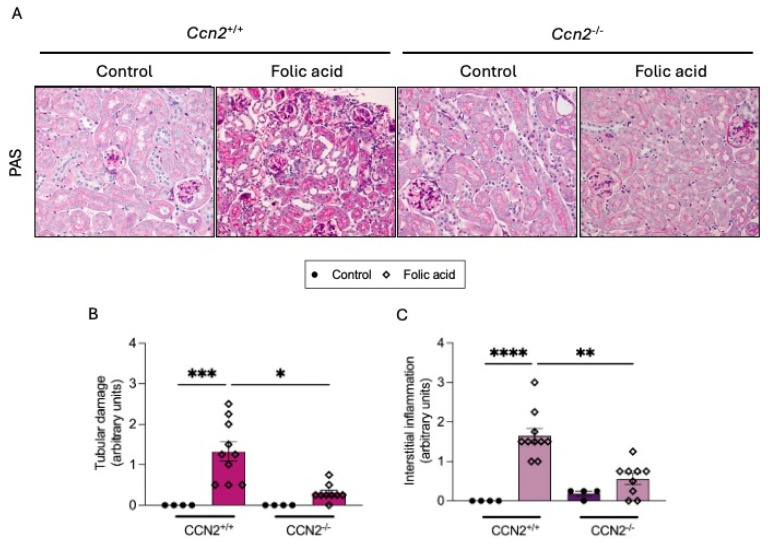
CCN2 deficiency prevents kidney morphological changes in chronic Folic Acid Nephropathy (CKD-FAN). CCN2flox/floxROSA26-ERT/Cre mice were treated with tamoxifen (10 mg/mL; i.p., 3 doses each 40 h) to obtain CCN2^−/−^ mice or with corn oil for the control group (CCN2^+/+^). After 2 weeks of washout, mice were injected with folic acid (FA; 250 mg/kg) or solvent, and studies were carried out after 3 weeks. Kidney tissues were obtained for histology and RNA studies. (**A**) Representative microphotographs of kidney sections stained with Periodic Acid-Schiff (PAS), showing the renal cortex from each group at 200× magnification. (**B**) Tubular damage and (**C**) interstitial inflammation were scored from 0 to 10 by evaluating tubular dilatation and necrosis, as well as perivascular and peritubular infiltration, represented as arbitrary units. Data are expressed as the average of 4 to 10 animals per group ± SEM. * *p* < 0.05, ** *p* < 0.005, *** *p* < 0.0005, **** *p* < 0.0001. The Kruskal–Wallis non-parametric statistical test was performed, followed by Dunn’s test without correction.

We have previously described that CCN2 deficiency diminished kidney damage in the acute phase of FAN (AKI-FAN), mainly by inhibiting necroinflammation and tubular cell death at 48 h [22]. CCN2 is also involved in the AKI-CKD transition phase, as shown by diminished inflammation and ECM upregulation observed at 7 days following the initial insult [20]. Here, we have further evaluated the role of CCN2 in the chronic phase of FAN; to this aim, mice were followed for 3 weeks. First, kidney morphological changes were evaluated by PAS staining. CCN2^+/+^ mice presented tubular damage and interstitial cell infiltration, which were significantly ameliorated in CCN2^−/−^ FAN mice (Figure 1A–C).

**Figure 2:**
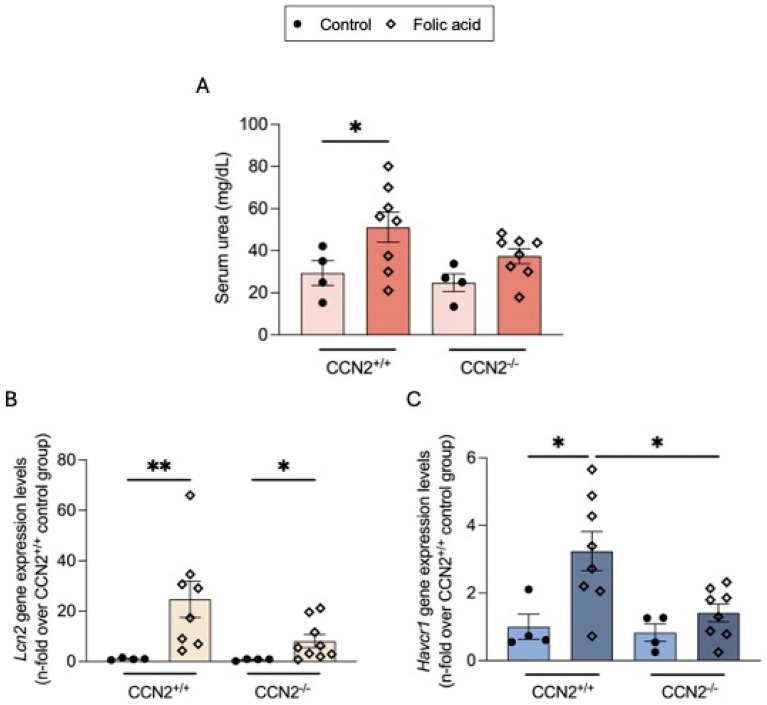
CCN2 deficiency diminished kidney damage biomarkers in chronic Folic Acid Nephropathy. CCN2^−/−^ and CCN2^+/+^ mice were injected with folic acid (FA; 250 mg/kg), and studies were carried out after 3 weeks. Kidney tissues were obtained for RNA studies. (**A**). Serum levels of urea are shown. (**B**) Lnc2 and (**C**) Havcr1 renal gene expression levels were measured by RT-qPCR and represented as n-fold. Data are expressed as the average of 4 to 10 animals per group ± SEM. * *p* < 0.05, ** *p* < 0.005. The Kruskal–Wallis non-parametric statistical test was performed, followed by Dunn’s test without correction.

Renal damage was confirmed by the evaluation of damage biomarkers. Serum urea was increased in FAN mice compared to controls (Figure 2A), indicating a persistent deterioration of kidney function in the chronic phase of the disease. Injured kidneys of FA-injected CCN2^+/+^ mice also presented increased renal gene expression of tubular stress and damage biomarkers Havcr1 and Lcn2, encoding the proteins KIM-1 and NGAL, respectively, as compared to controls. However, CCN2 deficiency caused a significant reduction in biomarkers of renal damage compared to wild-type mice (Figure 2B,C).

### 2.2. CCN2 Deficiency Diminishes Renal Inflammation and Fibrosis in Chronic Folic Acid Nephropathy

**Figure 3:**
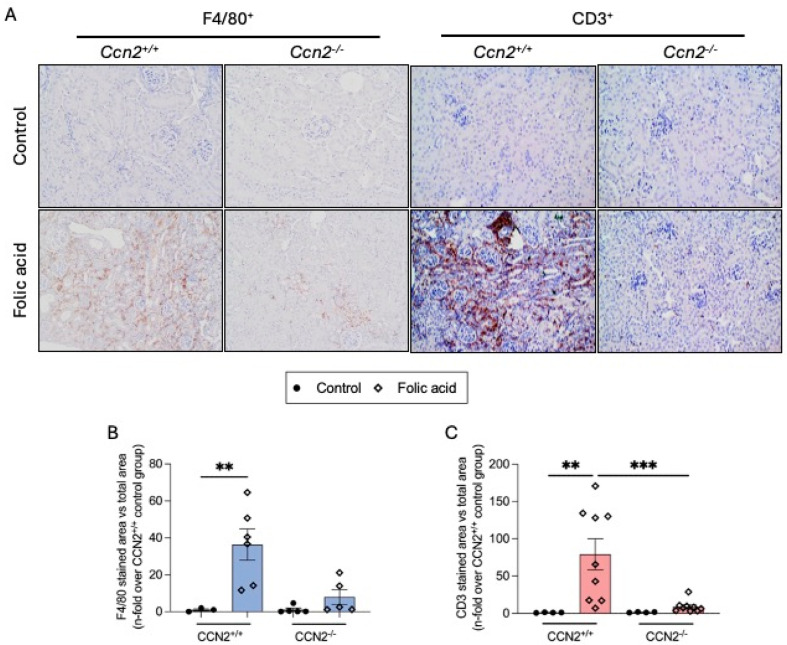
CCN2 deficiency reduced the number of inflammatory infiltrating cells in chronic Folic Acid Nephropathy. CCN2^−/−^ and CCN2^+/+^ mice were injected with folic acid (FA; 250 mg/kg), and studies were conducted after 3 weeks. Kidney immunohistochemistry was performed to evaluate the inflammatory cell infiltration. (**A**) Representative microphotographs of kidney sections showing the renal cortex from each group, with positive F4/80 staining (**left** panel) and positive CD3 staining (**right** panel), at 200× magnification. (**B**) Quantification of F4/80 and (**C**) of CD3 immunohistochemical staining expressed as mean stained area relative to the total area, represented as n-fold. Data are expressed as an average of 3 to 9 animals per group ± SEM. ** *p* < 0.005, *** *p* < 0.0005. One-way ANOVA statistical test followed by Fisher’s LSD test was performed.

CKD is characterized by sustained inflammation and increased collagen deposition, the latter a hallmark of fibrosis [4]. In the kidneys of CCN2-deficient mice, there was a significant decrease in the number of infiltrating macrophages (F4/80+ cells) and T lymphocytes (CD3+ cells) compared to the injured kidneys of CCN2^+/+^ mice 3 weeks after FA administration (Figure 3).

**Figure 4:**
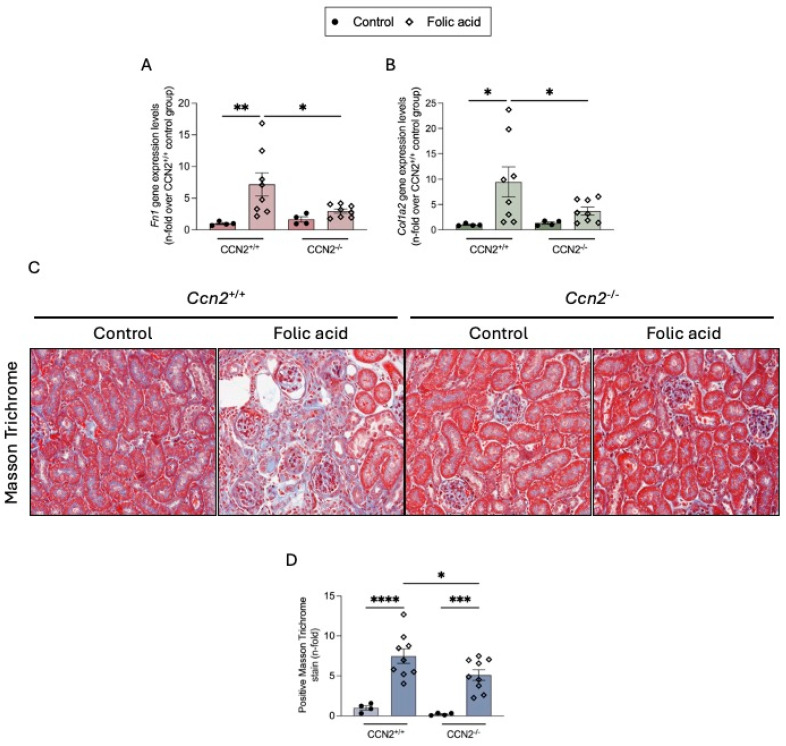
CCN2 deficiency markedly diminished ECM accumulation in chronic Folic Acid Nephropathy. CCN2^−/−^ and CCN2^+/+^ mice were injected with folic acid (FA; 250 mg/kg), and studies were conducted after 3 weeks. RNA was extracted from renal cortex. (**A**) Fn1 (Fibronectin) and (**B**) Col1a2 (Collagen I) gene expression levels were measured by RT-qPCR and represented as n-fold. (**C**) Representative Masson’s Trichrome-stained images of renal cortex sections from each group, shown at 200× magnification. (**D**) Quantification of blue staining indicating collagen accumulation, represented as n-fold. Data are expressed as an average of 4 to 10 animals per group ± SEM. * *p* < 0.05, ** *p* < 0.005, *** *p* < 0.0005, **** *p* < 0.0001. One-way ANOVA statistical test followed by Fisher’s LSD test was performed.

In CCN2^+/+^ FAN mice, increased renal gene expression levels of ECM components such as fibronectin (Fn1) and type I collagen (Col1a2) (Figure 4A,B) and collagen deposition, measured by Masson Trichrome staining (Figure 4C,D), were observed at 3 weeks. In CCN2^−/−^ FAN mice, renal Fn1 and Col1a2 gene expression was downregulated almost to control levels, and collagen accumulation was significantly diminished (Figure 4).

### 2.3. CCN2 Regulates Cellular Senescence in Chronic Folic Acid Nephropathy

In previous studies, we have described the role of CCN2 in modulating cellular senescence in the mouse model of IRI, but no studies in the nephrotoxic FAN model have been carried out.

First, we studied several markers of cellular senescence in the chronic phase of FAN, including DNA damage response (DDR), CCA, SASP, and antiapoptotic factors. All these markers have been involved in the activation of cellular senescence in renal damage [36].

**Figure 5:**
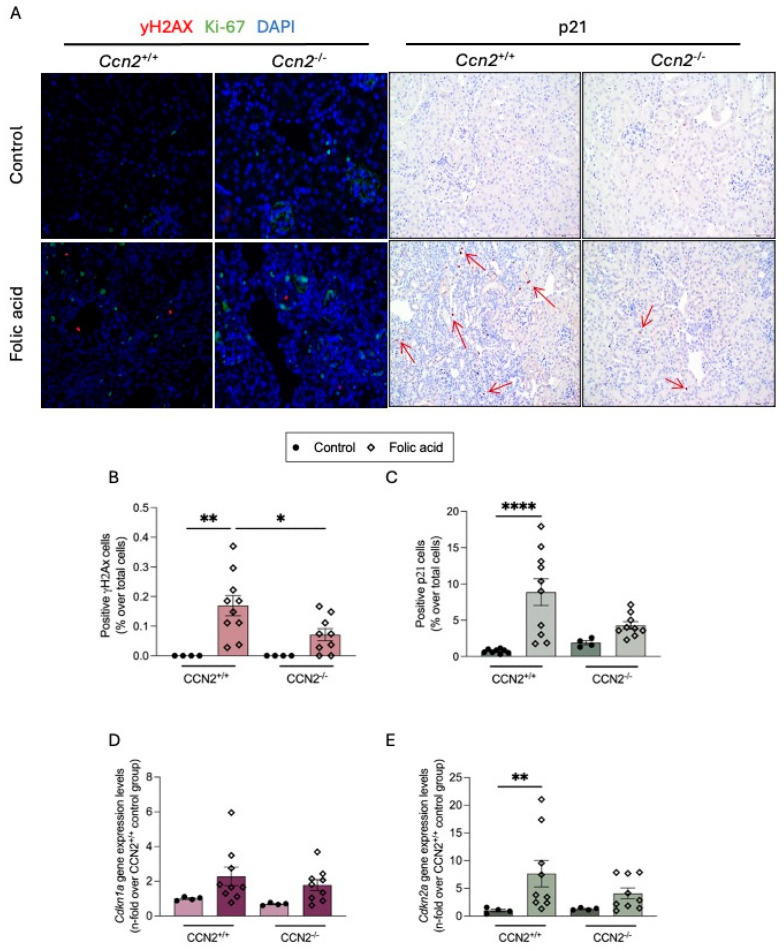
CCN2 deficiency diminished the DNA Damage Response and Cell-Cycle-arrest in chronic Folic Acid Nephropathy. CCN2^−/−^ and CCN2^+/+^ mice were injected with folic acid (FA; 250 mg/kg), and studies were carried out after 3 weeks. (**A**) Representative images of renal cortex sections from each group at 200× magnification. The right panel shows double immunofluorescence staining: γH2AX (red; Alexa 633), Ki-67 (green; Alexa 488), and nuclei counterstained with DAPI (blue). The left panel also shows immunohistochemistry of p21 cells per field. (**B**) Quantification of positive gH2AX nuclei and negative for Ki-67 per field and (**C**) quantification of positive p21 nuclei per field, represented as a percentage of positive vs. all nuclei. (**D**) Cdkn1a and (**E**) Cdkn2a gene expression levels were measured by RT-qPCR and represented as n-fold Data are expressed as average of 4 to 10 animals per group ± SEM. * *p* < 0.05, ** *p* < 0.005, **** *p* < 0.0001. One-way ANOVA statistical test followed by Fisher’s LSD test was performed for immunofluorescence staining, and the Kruskal–Wallis non-parametric statistical test followed by Dunn’s test without correction was performed for the rest of the data.

DDR was analyzed by immunohistochemistry, evaluating the levels of phosphorylated H2AX (γH2AX), a marker of the double-strand break [37]. Few positive nuclei for γH2AX (red) were found in FAN-injured kidneys. A key characteristic of senescent cells is cell cycle arrest. The nuclear Ki-67 protein (Ki-67) has previously been shown to be exclusively expressed in proliferating cells [38]. To note, all these DDR senescent-marker-positive cells (in red) were negative for the Ki-67 proliferative marker (in green; γH2AX^+^/Ki-67^−^), showing that DDR is only activated in non-proliferative, but not CCA, cells. Lower numbers of γH2AX-positive cells were observed in CCN2^−/−^ FAN kidneys compared to CCN2^+/+^ FAN kidneys, indicating reduced DDR in the absence of CCN2 following a nephrotoxic injury (Figure 5A,B).

The main characteristic of a senescent cell is prolonged CCA. In the kidneys of CCN2^+/+^ FAN mice, p21-positive nuclei were found in some tubular epithelial cells, whereas almost none was detected in CCN2^−/−^ FAN kidneys, similarly to what was observed in controls (Figure 5A,C). Some changes were also observed at the gene level; both *Cdkn1a* and *Cdkn2a* (which codify for p21 and p16, respectively) mRNA levels were upregulated in FAN kidneys compared to control kidneys (Figure 5D,E).

**Figure 6:**
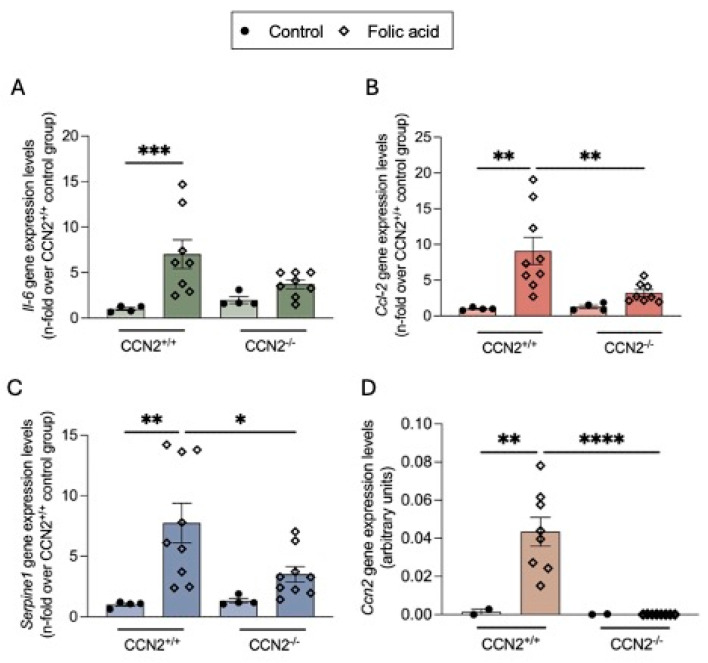
Renal overexpression of SASP components in chronic Folic Acid Nephropathy was prevented by CCN2 deficiency. CCN2^−/−^ and CCN2^+/+^ mice were injected with folic acid (FA; 250 mg/kg), and studies were conducted after 3 weeks. (**A**) Il-6, (**B**) Ccl-2, (**C**) Serpine-1, and (**D**) CCN2 gene expression levels in kidneys were measured by RT-qPCR and represented as n-fold. Data are expressed as the average of 4 to 10 animals per group ± SEM. * *p* < 0.05, ** *p* < 0.005, *** *p* < 0.0005, **** *p* < 0.0001. One-way ANOVA statistical test followed by Fisher’s LSD test was performed except for Il-6 expression, where the Kruskal–Wallis non-parametric statistical test followed by Dunn’s test without correction was performed.

Changes in SASP factor levels were evaluated by gene expression. In the kidneys of CCN2^+/+^ FAN mice, overexpression of the SASP cytokines Il6, Ccl2, Serpine1, and Ccn2 was observed compared to controls (Figure 6). By contrast, a decrease in gene expression levels of these cytokines was found in damaged kidneys of CCN2^−/−^ mice (Figure 6).

**Figure 7:**
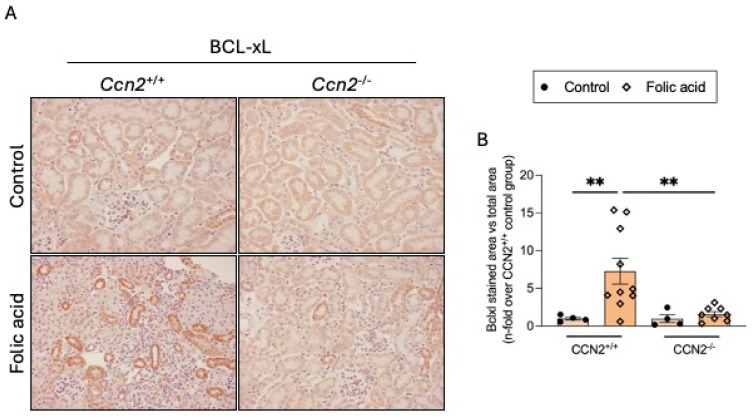
The antiapoptotic protein BCL-xL was increased in chronic Folic Acid Nephropathy but it is prevented by CCN2 deficiency. CCN2^−/−^ and CCN2^+/+^ mice were injected with folic acid (FA; 250 mg/kg), and studies were conducted after 3 weeks. (**A**) Representative immunohistochemical images of the renal cortex from each group, showing BCL-xL-positive staining at 200× magnification. (**B**) Quantification of BCL-xL immunohistochemical staining expressed as mean stained area relative to total area, represented as n-fold. Data are expressed as the average of 4 to 10 animals per group ± SEM. ** *p* < 0.005. A one-way ANOVA statistical test followed by Fisher’s LSD test was performed.

Another feature of senescent cells is the inhibition of apoptosis in senescent cells. The anti-apoptotic factor BCL-xL was found to be overexpressed in CCN2^+/+^ FAN mice in areas of tubular damage. However, BCL-xL renal levels in CCN2^−/−^ FAN mice were unaltered and similar to those observed in controls (Figure 7A,B).

### 2.4. CCN2 Regulates Cellular Senescence in the Acute Phase of Folic Acid Nephropathy

**Figure 8:**
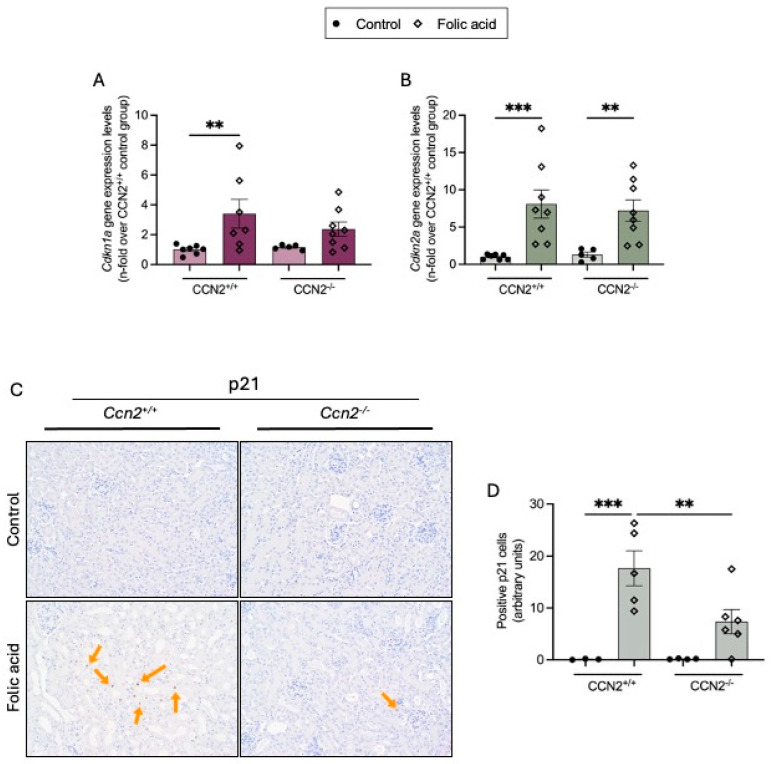
CCN2 deficiency diminished Cell-Cycle-Arrest in Acute Folic Acid Nephropathy (AKI-FAN). CCN2flox/floxROSA26-ERT/Cre mice were treated with tamoxifen (10 mg/mL; i.p., 3 doses each 40 h) to obtain CCN2^−/−^ mice or with corn oil in the control group (CCN2^+/+^). After 2 weeks of washout, mice were injected with folic acid (FA; 250 mg/kg) or solvent and studied at 48 h (**A**) *Cdkn1a* and (**B**) *Cdkn2a* gene expression levels were measured by RT-qPCR and represented as n-fold. (**C**) Micrographs of kidney sections from each group, showing positive p21 staining in the renal cortex at 200× magnification. (**D**) Quantification of positive p21 nuclei per field, represented as arbitrary units. Data are expressed as the average of 3 to 8 animals per group ± SEM. ** *p* < 0.005, *** *p* < 0.0005. One-way ANOVA statistical test followed by Fisher’s LSD test was performed except for *Cdkn1a* expression, where the Kruskal–Wallis non-parametric statistical test followed by Dunn’s test without correction was performed.

Activation of cellular senescence in the kidney has been described in several experimental AKI models [39,40,41]. Therefore, we further evaluated the protective effects of CCN2 deficiency on senescent markers at 48 h in experimental FAN in the acute phase of renal damage induced by exposure to this nephrotoxic compound. In kidneys of FAN CCN2^+/+^ mice, increased *Cdkn1a* and *Cdkn2a* gene expression compared to controls was found (Figure 8A,B). Moreover, the presence of senescent cells in FAN kidneys was detected by IH, as shown by positive p21 immunostained cells, mainly tubular cells. Importantly, CCN2 deficiency significantly reduced the number of p21-positive cells in FAN kidneys (Figure 8C).

**Figure 9:**
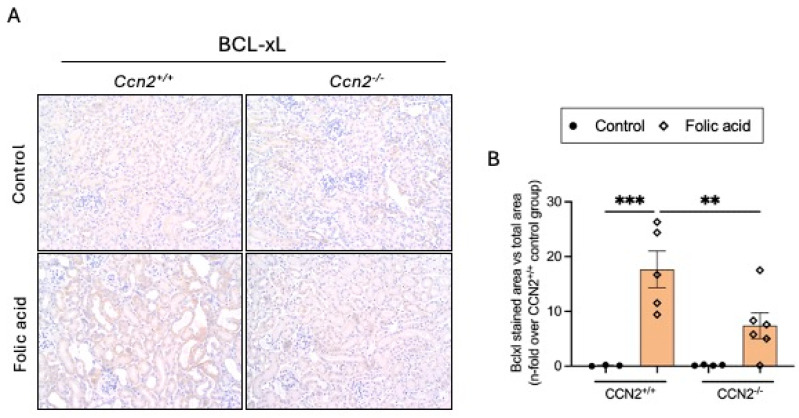
CCN2 deficiency diminished the antiapoptotic protein BCL-xL in acute Folic Acid Nephropathy. (**A**) Images of kidney tissue from each group, showing the renal cortex with BCL-xL-positive staining at 200× magnification. (**B**) Quantification of BCL-xL immunohistochemical staining expressed as mean stained area relative to the total area, represented as n-fold. Data are expressed as the average of 4 to 10 animals per group ± SEM. ** *p* < 0.005, *** *p* < 0.0005. A one-way ANOVA statistical test followed by Fisher’s LSD test was performed.

In AKI-FAN, at 48 h following FA administration, protein expression of BCLxL was increased in CCN2^+/+^ mice, while it was significantly reduced in CCN2^−/−^ mice (Figure 9A,B).

In this model, we previously observed less inflammation and diminution of renal proinflammatory factors, including SAPS genes in CCN2-deficient kidneys compared to control kidneys [22]. The present finding in CCN2-deficient mice, including the diminution of the number of senescent cells in injured kidneys and the downregulation of senescent markers, suggests that CCN2 may play a determinant role in the initial events of FAN leading to the activation of senescence in the kidney.

### 2.5. CCN2 Deficiency Prevents the Loss of the Nephroprotective Factor Klotho in Acute and Chronic Folic Acid Nephropathy

**Figure 10:**
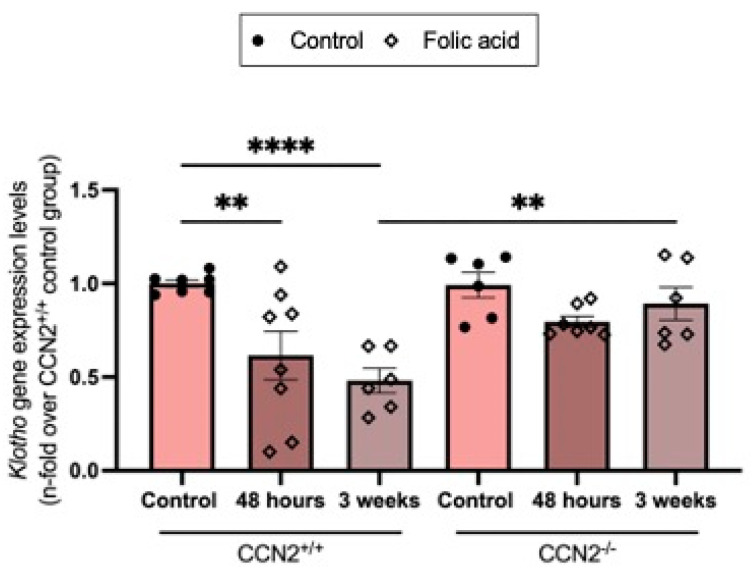
CCN2 deficiency prevented the loss of Klotho in acute and chronic Folic Acid Nephropathy. *Klotho* gene expression levels were measured by RT-qPCR and represented as n-fold. Data are expressed as average of 4 to 10 animals per group ± SEM. ** *p* < 0.005, **** *p* < 0.0001. One-way ANOVA statistical test followed by Fisher’s LSD test was performed.

Previous studies have described a downregulation of the nephroprotective factor Klotho in human and experimental models of AKI and CKD [42]. The administration of FA to CCN2^+/+^ mice decreased renal *Klotho* gene expression levels at 48 h and further decreased them at three weeks. However, in CCN2-deficient mice, the loss of *Klotho* gene expression levels was prevented, showing levels were similar to controls (Figure 10). This indicates that CCN2 plays a protective role in maintaining Klotho expression during FAN.

### 2.6. CCN2 Deficiency Prevents Capillary Rarefaction in Chronic Folic Acid Nephropathy

**Figure 11:**
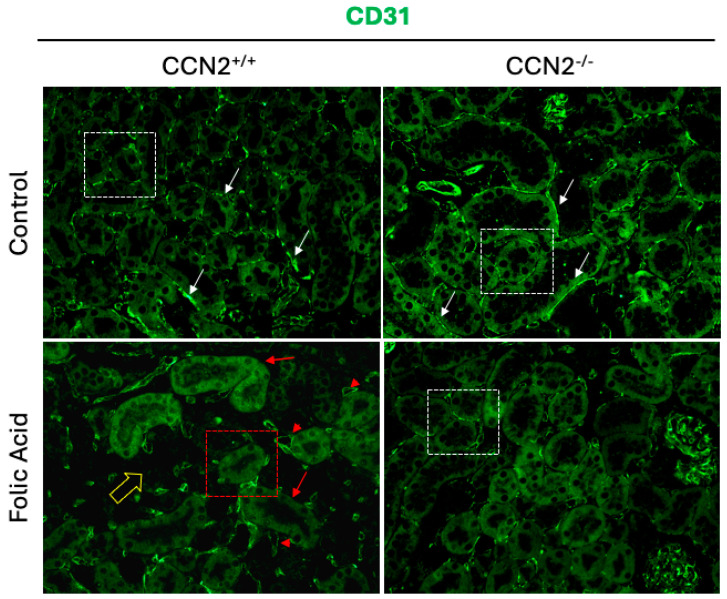
CCN2 deficiency diminished renal rarefaction in chronic Folic Acid Nephropathy. CCN2^+/+^ and CCN2^−/−^ mice were injected with folic acid (FA; 250 mg/kg) or solvent (control) and studied after 3 weeks. Immunofluorescence of the endothelial marker CD31 in kidneys was marked with Alexa 488 (green). The figure shows representative images of 4 to 10 animals per group at 400× magnification.

Peritubular capillary rarefaction has been identified as a significant indicator of CKD. This phenomenon has been demonstrated to play a pivotal role in CKD progression and fibrosis. The underlying mechanism involves the impairment of nutrient and oxygen delivery to tubular cells, which in turn leads to enhanced kidney injury [43,44,45]. Analysis of capillaries by CD31 staining showed that CCN2^−/−^ and CCN2^+/+^ control mice displayed an extensive microcapillary network, which is characterized by its continuity along the tubules (white arrows and boxed tubules as examples) (Figure 11). In CCN2^+/+^ FAN mice, microvascular rarefaction was found, showing loss of microvessel density and ramifications (red arrows and boxed tubules as representatives) in areas of fibrosis or in adjacent tubules (yellow arrow), as well as concentration of the positive green mark in dilated capillaries (red arrowheads) (Figure 11). However, CCN2 deficiency prevented the FAN-induced capillary rarefaction in the renal cortex, showing a preserved endothelial network surrounding the tubules (representative boxed tubule) (Figure 11).

### 2.7. CCN2 Induces Cellular Senescence in HUVECs

**Figure 12:**
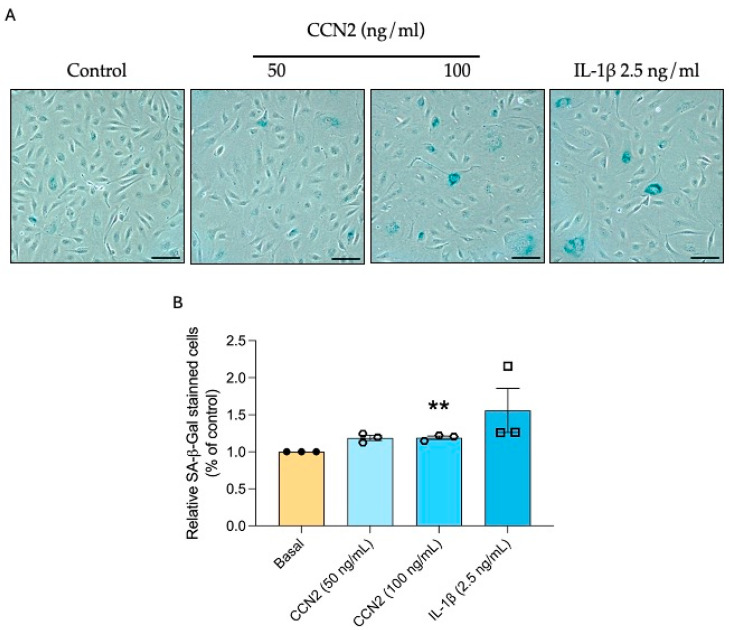
CCN2 induces senescence-associated β-galactosidase activity in a primary culture of Human Umbilical Vein Endothelial Cells (HUVEC). Primary cultures of HUVEC isolated from donor human umbilical cords were stimulated for 24 h with 50 ng/mL or 100 ng/mL recombinant CCN2 or 2.5 ng/mL IL1β used as a positive control. (**A**) Representative images and (**B**) quantification of the percentage of cells stained with SA-β-galactosidase versus total. Scale bar = 50 μm. These data are shown as percentages and presented as the mean ± SEM of 4 independent experiments. ** *p* < 0.005. Student’s *t*-test was used for independent samples.

**Figure 13:**
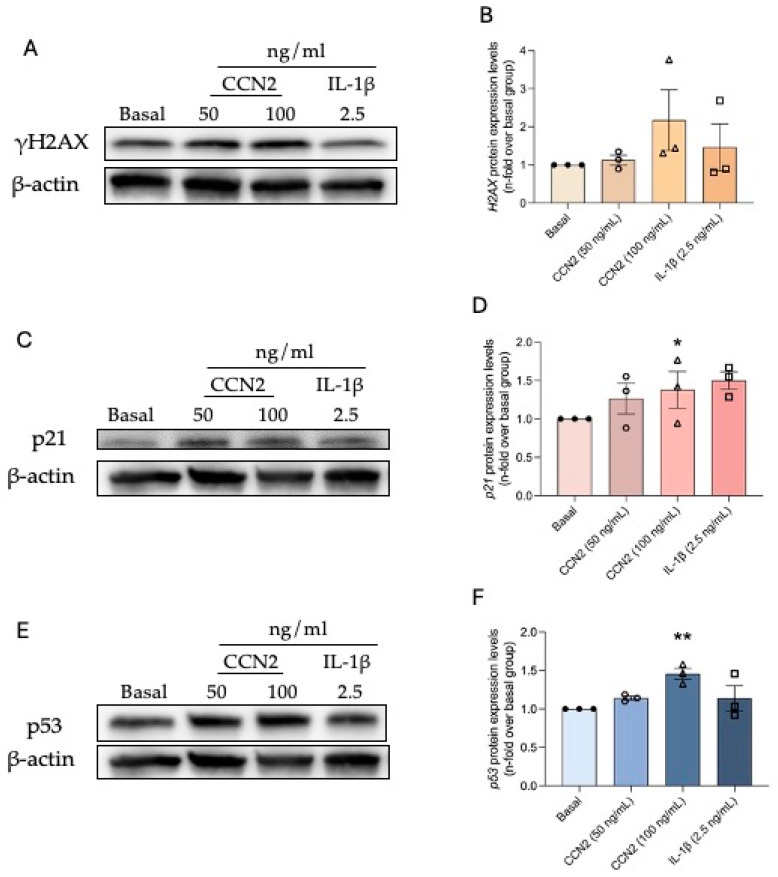
CCN2 induces protein expression of senescence markers in a primary culture of human umbilical vein endothelial cells (HUVEC). Primary cultures of HUVEC were stimulated for 24 h with 50 ng/mL or 100 ng/mL recombinant CCN2 or 2.5 ng/mL IL-1β used as a positive control. The protein levels of (**A**) γH2AX, (**C**) p21, and (**E**) p53 were determined by Western blot, using β-actin as a loading control. Representative blot images are shown on panels (**B**,**D**,**F**). Results are shown as n-fold and are presented as mean ± SEM of 3 independent experiments. * *p* < 0.05 and ** *p* < 0.005 by Student’s *t*-test used for independent samples.

Since CCN2 induces tubular senescence and endothelial damage (capillary rarefaction), we evaluated whether CCN2 has a direct role in endothelial senescence. To this aim, senescence-associated β-galactosidase activity was assessed in primary human umbilical vein endothelial cells (HUVEC). Stimulation of HUVECs with 50 or 100 ng/mL of recombinant CCN2 increased SA-β-gal activity (Figure 12). In addition, CCN2-induced DDR and CCA were studied, observing an increase in the protein expression of γH2AX, p21, and p53 (Figure 13).

### 2.8. CCN2 Induces the Priming and Activation of the NLRP3 Inflammasome in HUVECs

**Figure 14:**
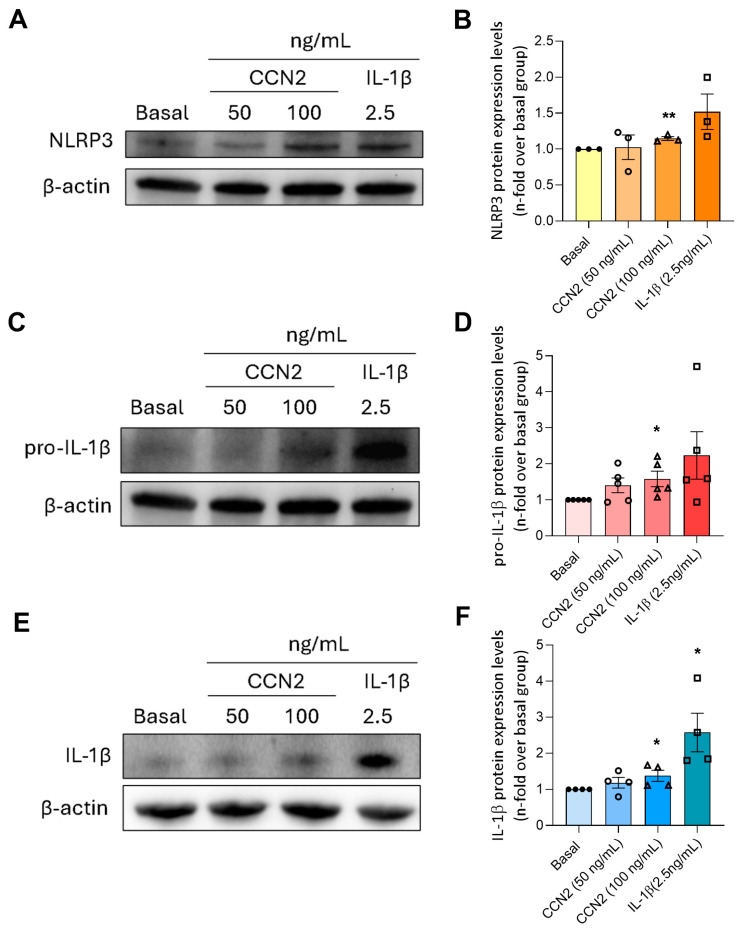
CCN2 induces the priming and activation of the NLRP3 inflammasome in human umbilical vein endothelial cells (HUVEC). Primary cultures of HUVEC were stimulated for 24 h with 50 ng/mL or 100 ng/mL recombinant CCN2 or 2.5 ng/mL IL1β used as a positive control. The protein levels of (**A**) NLRP3, (**C**) pro-IL1β, and (**E**) mature IL1β were determined by Western blot, using β-actin as a loading control. Representative blot images are shown on panels (**B**,**D**,**F**). Results are shown as n-fold and are presented as mean ± SEM of 3–5 independent experiments. * *p* < 0.05 and ** *p* < 0.005 by Student’s *t*-test used for independent samples.

Next, we evaluated if CCN2 could promote the priming and activation of the NLRP3 inflammasome, which has been previously reported as an inductor of cellular senescence in endothelial cells [46,47,48] and other cell types [49,50]. After treatment with 100 ng/mL of CCN2, the protein levels of NLRP3 and pro-IL1β, used as markers of the NLRP3 inflammasome priming, were increased (Figure 14A, B). Additionally, the mature form of IL-1β, a main end-product of the inflammasome activation, was also enhanced by CCN2 (Figure 14C).

### 2.9. CCN2 Modulates the NLRP3 Inflammasome in Chronic Folic Acid Nephropathy

**Figure 15:**
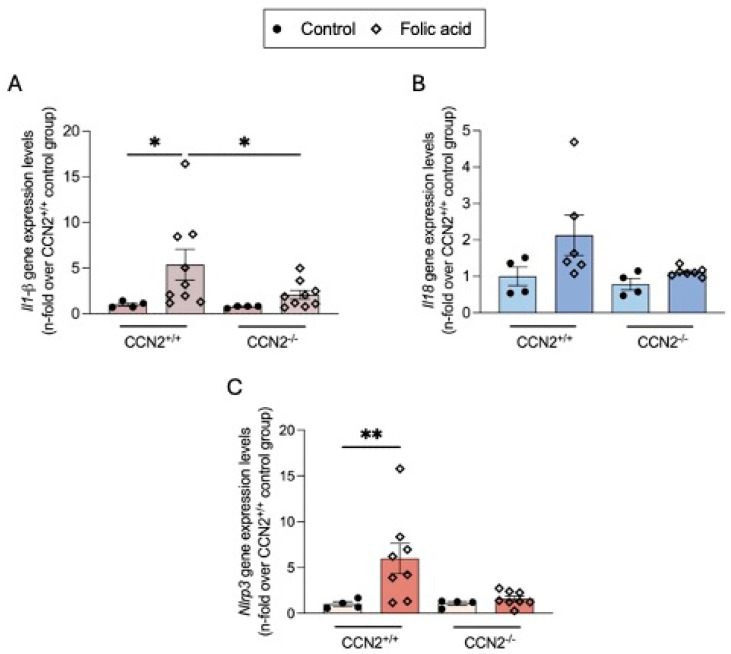
CCN2 deficiency diminished the gene expression of inflammasome components in chronic Folic Acid Nephropathy. (**A**) Kidney gene expression of (**A**) *Il-1b,* (**B**) *Il-18,* and (**C**) *Nlrp3* were measured by RT-qPCR and represented as n-fold. Data are expressed as average of 4 to 10 animals per group ± SEM. * *p* < 0.05, ** *p* < 0.005. One-way ANOVA statistical test followed by Fisher’s LSD test was performed.

In the AKI phase of FAN, CCN2 is involved in the activation of the NLRP3 inflammasome pathway [22]. Now, we have observed that in the chronic phase of FAN, gene expression levels of the *Nlrp3* and its effectors *Il-18* and *Il-1β* were also found upregulated in injured kidneys compared to controls, whereas in CCN2^−/−^ FAN their expression levels were downregulated (Figure 15).

## 3. Discussion

Our preclinical studies using a total conditional CCN2 knockout mouse model demonstrate that CCN2 plays a significant role in the development of a senescence phenotype after exposure to a nephrotoxic agent, both in the early and in the chronic phase of FAN. The identification of positive senescent TECs in chronic FAN and its absence in CCN2-deficient mice support the key role of CCN2 in the induction of tubular senescence, secondary senescence, and renal fibrosis. Moreover, the in vitro studies in primary cultures of endothelial cells demonstrate that CCN2 directly activates cellular senescence in these cells. In addition, the prevention of vascular rarefaction in CCN2-deficient mice supports that CCN2 can contribute to endothelial damage leading to endothelial senescence and subsequent kidney damage progression.

Several lines of evidence suggest that CCN2 plays an important role in the regulation of cellular senescence. CCN2 has been identified as a key component of SASP [27]. This growth factor can be released by senescent cells, contributing to secondary senescence and therefore amplifying tissue damage. Indeed, in numerous preclinical models, tissue CCN2 levels were elevated, and the presence of senescent cells was identified [19,27,36], as observed here in acute and chronic FAN. In addition, CCN2 overexpression was associated with senescent TECs in human allograft rejection [51]. Some in vitro studies have demonstrated that CCN2 can also directly induce cellular senescence, as observed in primary cultured TECs [19] and fibroblasts [27,52,53]. In the present study, we described that CCN2 directly induces cellular senescence in primary cultures of human endothelial cells. Previous studies have also shown that CCN2 induces CCA in mesangial and bronchial endothelial cells [52,53], indicating that in other cells CCN2 could induce a senescent phenotype in different pathological conditions.

Microvessels consisting of endothelial cells and pericytes form intricate networks in the kidney microvasculature [54]. In aging mice and human kidneys, microvascular rarefaction and reduced numbers of pericytes have been linked to renal function decline [55]. Accordingly, in CKD, microendothelial damage and pericyte detachment are associated with renal fibrosis and dysfunction [56,57,58]. Vascular rarefaction is not only a characteristic of fibrotic tissues, including the kidney, but also a driver of kidney damage progression [59]. Vascular rarefaction leads to endothelial cell dysfunction, phenotype changes to endothelial-to-mesenchymal transition, and premature senescence of endothelial cells [60]. Rarefaction is an intrinsic characteristic of different aged tissues, as demonstrated in the failing heart by a correlation between this phenomenon and the accumulation of p53-positive endothelial cells [61]. Moreover, in a recent study, endothelial-specific deletion of p53 reverses the deleterious vascular phenotype and improves hyperoxia-induced damage [62]. Our data in CCN2 knockout mice demonstrated a significant prevention of vascular rarefaction and senescence markers in injured kidneys, suggesting that CCN2 can regulate both processes to contribute to renal damage progression. Our in vitro studies demonstrated that CCN2 increased senescence markers, such as γH2AX, p21, and p53 protein expression, and induced SA-β-gal activity in primary cultured human endothelial cells, clearly showing that CCN2 can induce endothelial senescence. Previous studies have demonstrated that CCN2 causes endothelial dysfunction in ex vivo isolated aortic rings and in mice [63], supporting these findings. In addition, CCN2 is a critical paracrine regulator of microvascular integrity [64] and participates in endothelial basement membrane formation and angiogenesis [65]. Human skin biopsies of patients with systemic sclerosis presented elevated CCN2 levels associated with phenotype changes in endothelial cells and induction of senescence markers in fibroblasts. This suggests that damaged endothelial cells can produce CCN2, which acts in a paracrine manner to induce cellular senescence in fibroblasts, contributing to skin fibrosis [66]. Furthermore, studies of gain-and-loss of function using endothelial cell-specific overexpression or deletion of *Sox9* support that CCN2, a direct SOX9 target, could be released by endothelial and activate fibroblasts to promote fibrosis [67]. Many studies have described activation of the NLRP3 inflammasome pathway in response to kidney damage. However, previous data suggested that NLRP3 inflammasome activation in TECs may be limited or even absent, whereas other cells, such as dendritic cells or macrophages, play a key role in this signaling system [68]. Importantly, CCN2 directly activated the NLRP3 inflammasome complex in human endothelial cells and thus the formation of mature IL-1b. This is of relevance since the CANTOS clinical trial demonstrated that this pro-inflammatory cytokine is directly involved in the development of human vascular disease [69]. In fact, it is itself a promoter of premature senescence in human endothelial cells [46,47]. Taken together, CCN2 could induce microvascular destabilization, endothelial senescence, and, therefore, contribute to renal damage progression. Since CCN2 is itself a component of the SASP, this factor could be key in perpetuating and amplifying cell senescence and tissue damage in an autocrine or paracrine loop.

Many studies have demonstrated activation of cellular senescence, both in AKI, in the AKI-to-CKD transition phase, and in CKD [70]. Our data in experimental FAN confirm these findings. In the AKI-FAN, we have found that senescence was activated in injured TECs, showing an increased DNA damage response and p21 nuclear expression, a marker of CCA, as well as elevated kidney expression of SASP genes. Furthermore, senescent TECs and SASP overexpression remained elevated in the chronic phase of FAN. Studies carried out here in CCN2-deficient mice in the FAN model clearly showed that CCN2 absence significantly diminished senescent markers associated with lower inflammation (including inflammasome activation) and fibrosis. Several studies support that maladaptive repair can lead to senescent TECs, characterized by CCA and overproduction of SAPS, leading to kidney damage progression [71,72,73]. Our findings clearly show that CCN2 deficiency protects against renal fibrosis induced by a nephrotoxic agent, in part by inhibition of cellular senescence.

Another potential mechanism by which CCN2 may contribute to the progression of renal injury is through secondary senescence. Senescent cells produce an aberrant secretome (the SASP) that can act as a paracrine signaling mediator on neighboring cells, promoting inflammation, fibrosis, and spreading damage and senescence [74,75]. The SASP genes are regulated by several transcription factors, including NF-κB, one of the most important and best studied [74]. CCN2 activates the NF-κB pathway in the kidney and in several in vitro renal cell types, resulting in the upregulation of gene expression of numerous SASP components, including proinflammatory cytokines, such as IL-6 or CCL-2 [16]. In this sense, in FAN renal, activation of NF-κB and overexpression of related proinflammatory cytokines were described [22,76,77] coincidentally with the same time points of *Ccn2* gene upregulation observed in the present study. Moreover, CCN2 deficiency prevented the renal overexpression of SASP genes induced by FAN. All these results support that CCN2 drives renal damage progression through activation of NF-κB/SASP/secondary senescence.

The present data confirm and extend previous studies in CCN2-deficient mice, demonstrating the key role of CCN2 in renal fibrosis following IRI [19,31], FAN [20], and chronic graft dysfunction [51,78]. Indeed, CCN2 has been proposed as a surrogate marker of fibroproliferative disorder for several conditions [79]. Moreover, circulating or urinary CCN2 protein or degradation products can be a potential disease biomarker for human CKD [4,25,80]. Moreover, CCN2 deficiency also decreased inflammation-related events in the acute phase of the IRI model through a reduction in the oxidative stress response [29] and in AKI-FAN by diminution of necroinflammation [22]. The acute phase of FAN is characterized by tubular damage, cell death, and inflammation [81,82,83,84]. Previous studies have demonstrated that CCN2 can activate inflammation-associated cell death pathways, such as necroptosis and ferroptosis [22]. Now, we have confirmed and extended previous observations describing that CCN2 deficiency reduces the inflammatory response in FAN, as evidenced by a decrease in the recruitment of T cells and macrophages, as well as decreased inflammasome activation in acute and chronic phases. Altogether, these data demonstrate the complexity of CCN2s role in the regulation of pathological processes associated with kidney damage. Collectively, they suggest that reduced autocrine and paracrine induction of senescence may, at least in part, explain the association between reduced renal damage and inflammation and reduced senescent cell accumulation.

Klotho is an anti-aging protein highly expressed in the normal tubular epithelium, and its loss is considered an early event in AKI [85]. The results of this preclinical study demonstrate that CCN2 deficiency can prevent the loss of Klotho both in the acute and chronic phases of FAN. Renal tubular-specific depletion of klotho is associated with a predisposition to renal fibrogenesis, implicating epithelial klotho loss in renal disease progression [86]. The recovery of Klotho, through recombinant protein delivery or plasmid expression, attenuated experimental renal fibrosis induced by AKI or CKD, demonstrating the therapeutic efficacy of Klotho restoration [87]. Other studies have corroborated an inverse relation between Klotho and another SASP component, PAI-1. In renal epithelial cells, PAI-1 overexpression promotes epithelial dysfunction (as evidenced by dedifferentiation, CCA, and induction of SASP factors, including CCN2) and Klotho loss, whereas restoration of Klotho levels decreased *Ccn2* expression [88].

Experimental studies have demonstrated that the selective elimination of senescent cells (using senolytics) or altering the SASP program may serve as potential therapeutic strategies against aging [89]. Recent data support that suppression of pathological SASP secretion (using senomorphics), without killing senescent cells, should be a safer alternative to senolytics and a better therapeutic option [90]. In this sense, anti-CCN2 therapy, which is currently being investigated in phase 2 and phase 3 trials of different diseases [91], may be used to reduce acute and chronic renal dysfunction following FAN by limiting the accumulation of senescent cells (as a senomorphic). Consequently, anti-CCN2 treatments may prove beneficial to treat AKI and prevent progression to CKD.

These findings provide strong evidence regarding the crucial role of CCN2 in senescence activation, highlighting how both cell cycle arrest and SASP have a pivotal role in kidney injury progression. Although the CCN2 deficiency has been observed to be beneficial in renal disease, it may be deleterious in other tissues and conditions. For instance, CCN2 deficiency predisposed to the formation of aortic aneurysms [31], whereas cardiac-specific overexpression of CCN2 and postischemic administration of recombinant CCN2 protected from acute myocardial IRI [92]. This phenomenon may be explained by the context-dependent beneficial antifibrotic function of cellular senescence under specific conditions, including cutaneous wound healing and liver injury [93,94], illustrating some limitations of the present study.

## 4. Materials and Methods

### 4.1. Animals

Experiments were performed according to the European Community guidelines for animal experiments and the ARRIVE guidelines and with the consent of the Experimental Animal Ethics Committee of the Health Research of the IIS-Fundación Jiménez Díaz and PROEX065/18 and PROEX 242.2/21 of the Comunidad de Madrid. Animals were sacrificed with an overdose of CO_2_ in a special chamber. Blood and urine were collected, and kidneys were perfused in situ with saline before removal. Half of each kidney (2/4) was fixed, embedded in paraffin, and used for immunohistochemistry, and the rest was snap-frozen in liquid nitrogen for renal cortex RNA and protein studies.

Conditional CCN2 deficient mice: The generation of time-conditional CCN2 complete knockout mice, homozygous CCN2 flox mice (generated by Dr. Andrew Leask’s laboratory; University of Western Ontario, Canada [95,96]), was crossed with tamoxifen-inducible Cre recombinase (CreERT2) mice under the control of the ROSA26 locus (ROSA26CreERT2; The Jackson Laboratory, Bar Harbor, ME, USA). ROSA26CreERT2 and CCN2 flox mice, both with a C57Bl/6J genetic background, were crossbred for five generations, and homozygous CCN2/floxROSA26-ERT/Cre mice were used in our experimental design described in this manuscript. The genotype of mice was confirmed with polymerase chain reaction (PCR) using the following primers: CCN2 flox forward (5′-AAAGTCGCTCTGAGTTGTTAT-3′) and reverse (5′-CCTGATCCTGGCAATTTCG3′); ROSA26CreERT2 forward (5′-AATACCAATGCACTTGCCTGGATGG3′) and reverse (5′-GAAACAGCAATTACTACAACGGGAGTGG-3′); and (5′-GGAGCGGGAGAAATGGATATG-3′). CCN2 gene deletion in male mice CCN2/floxROSA26-ERT/Cre was induced with the i.p. administration of 4 injections of 10 mg/mL tamoxifen (resuspended in corn oil) over 7 days (CCN2^−/−^ mice). Control mice were injected with corn oil (CCN2^+/+^ mice). After a two-week washout period (counting from the last injection) was developed, and then CCN2 deletion was confirmed with polymerase chain reaction (PCR) using CCN2-floxed Forward: 5′-AATACCAATGCACTTGCCTGGATGG-3′ and CCN2-floxed Reverse: 5′-GAAACAGCAATTACTACAACGGGAGTGG-3′ primers. The amplified DNA was resolved by size in agarose gels (1.5%). Tamoxifen-induced recombination resulted in a 77% reduction in CCN2 mRNA.

Folic acid nephropathy (FAN) is a classical model of kidney tubulointerstitial injury characterized by acute renal failure, tubular cell death, interstitial leukocyte infiltration, and subsequent tubular regeneration [76] that has been reported in humans [97]. CCN2^+/+^ or CCN2^−/−^ mice were divided into two additional groups: one received a single FA intraperitoneal injection (250 mg/kg; Sigma-Aldrich, St. Louis, MO, USA) in 0.3 mol/L sodium bicarbonate (n = 4–10 animals), and the other received vehicle (n = 4–10 animals). Mice were sacrificed 48 h later, at the acute phase of AKI characterized by cell death and an inflammatory response in the kidney [22], or at 3 weeks, in the chronic phase of renal damage.

### 4.2. Cell Cultures

Primary Human Umbilical Vein Endothelial Cells (HUVECs) were obtained by collagenase digestion from umbilical cords provided by Hospital Universitario La Paz from donors with informed consent, cultured as described previously [98] using 50 ng/mL or 100 ng/mL recombinant CCN2 (Peprotech, 120-19). IL1β 2.5 ng/mL (Prepotech, 200-01B) was used as a positive control for senescence.

### 4.3. Senescence-Associated-β-Galactosidase Activity

Cells were seeded into 6-well plates at a density of 50,000 cells/well and were exposed to different stimuli. After 24 h, the senescence-associated-β-galactosidase (SA-β-gal) activity was measured using the Senescence Cells Histochemical Staining Kit (CS0030, Sigma-Aldrich, St. Louis, MO, USA), as previously described [99]. The ratio of SA-β-gal positive cells/total cells was calculated by treatment-blinded manual counting of at least 1000 total cells in 12 random fields under phase contrast illumination, using an inverted microscope EVOS M5000 imaging system (Invitrogen, Waltham, MA, USA).

### 4.4. Cellular Protein Studies

Total proteins were isolated from cells seeded at a density of 200,000 cells/60 mm plate. After 24 h of stimulation, samples were collected by mechanical scraping with ice-cold lysis buffer and quantified using a BCA protein assay kit (A65453, ThermoFisher, Waltham, MA, USA). Proteins (20 μg) were separated on 8–15% acrylamide gels using SDS-PAGE, as described [36]. After electrophoresis, samples were transferred to polyvinylidene difluoride membrane (Millipore, Burlington, MA, USA), blocked in TBS containing 0.1% Tween 20 and 5% dry non-fat milk for 1 h at room temperature, and incubated in the same buffer with different primary antibodies overnight at 4 °C. After washing, membranes were incubated with the appropriate HRP (horseradish peroxidase)-conjugated secondary antibody (Invitrogen) for 1 h at room temperature and developed using an ECL kit (Amersham Biosciences). Results were analyzed by LAS 4000 and Amersham Imager 600 (GE Healthcare, Chicago, IL, USA) and quantified by densitometry using Quantity One software 4.6.6 (Biorad, Hercules, CA, USA). The following primary antibodies were employed (dilution): anti-CDKN1A/p21 (1:500, Santa Cruz, sc-6246), anti-p53 (1:1000, Santa Cruz, sc-126), Phospho-Histone H2AX (1:1000, Cell Signaling, #2577), NLRP3 (1:1000, Adipogene, #20B-0012), and IL-1β/IL-1F2 (1:1000, R&D System, #AF-401-NA); mouse monoclonal anti–β-actin antibody (1:50,000; A3854; Sigma-Aldrich) was used as a loading control.

### 4.5. Histology and Immunohistochemistry

Paraffin-embedded kidney sections were stained using standard histology procedures, as described elsewhere [100]. Periodic acid-Schiff (PAS, Sigma-Aldrich) stained slides were quantified, assessing tubular damage as tubular dilation and interstitial inflammatory infiltrate as arbitrary units as previously described [101]. Masson’s trichrome staining standard procedure was used (Dako, Hovedstaden, Denmark). Slides were quantified using Image Pro-plus Software 4.5.0, determining the positive red staining area relative to the total area.

Immunohistochemistry (IH) and immunofluorescence (IF) were performed on 3 μm thick tissue sections. For IH, antigens were retrieved using the PTlink system (DAKO) with sodium citrate buffer (10 mM) adjusted to pH 6–9, depending on the immunohistochemical marker. Endogenous peroxidase was blocked. Sections were incubated for 1 h at room temperature with Casein Solution (Vector Laboratories, Newark, CA, USA) to remove non-specific protein binding sites. Then, primary antibodies were incubated overnight at 4 °C. Specific HRP-conjugated (Dako, Hovedstaden, Denmark) or biotinylated secondary antibodies (Amersham Biosciences) were used. The latter was followed by Avidin-Biotin Complex incubation (Vector Laboratories). Signal was developed with a substrate solution and 3,3-diaminobenzidine as a chromogen (Abcam, Cambridge, UK). Finally, slides were counterstained with Carazzi’s hematoxylin (Richard Allan Scientific, Kalamazoo, MI, USA).

For double immunofluorescence evaluation, slides were subjected to peroxidase-blocking solution for 30 min followed by antigen retrieval by boiling in pH6 citrate buffer. First, α-Ki67 was incubated ON at 4 °C, followed by α-rabbit HRP ready-to-use for 45 min and then incubated with Opal^TM^ Dye 520 (PerkinElmer, Waltham, MA, USA) diluted 1:200 for 10 min. After this, a second antigen retrieval was performed, and slides were incubated with the gH2AX primary antibody for 1.5 h and then with Alexa Fluor 555 diluted 1:100 for 45 min. For single immunofluorescence, evaluation slides were subjected to antigen retrieval by boiling in pH 9 citrate buffer. Sections were incubated for 1 h at room temperature with PBS, 4% BSA, and 10% horse serum to remove non-specific protein binding sites. Then, primary antibodies were incubated overnight at 4 °C. Specific Alexa Fluor 488 secondary antibody was used. Nuclei were counterstained with DAPI, and coverslips were mounted with ProLong^TM^ Gold antifade reagent (Invitrogen). The primary antibodies used were [dilution]: P21 ([1:2000, Ab188224, Abcam), gH2AX ([1:500], NB1002280 Novus Biological, Cambridge, UK), BCL-xL ([1:4000], ab178844, Abcam), Ki-67 ([1:200], Thermofisher RM9106S), anti–F4/80 (1:50; reference MCAP497; Bio-Rad), anti–CD3 (ready to use; Dako), and anti-CD31 ([1:30], DIA-310-BA-2, Dianova, Geneva, Switzerland).

Quantification was made by using the Image-Pro Plus software version 7.1 to determine the positive staining area relative to the total area or counting positive staining manually (in the case of P21 immunohistochemistry) in 5–10 randomly chosen fields (×200 magnification).

### 4.6. Gene Expression Studies

RNA from the renal cortex was isolated with TRItidy G^TM^ (PanReac; Barcelona, Spain). cDNA was synthesized by a High-Capacity cDNA Archive kit (Applied Biosystems, Foster City, CA, USA) using 2 μg of total RNA and following the manufacturer’s instructions. Quantitative gene expression analysis was performed on a QuantStudio™ 3 fast real-time PCR system (Applied Biosystems) using fluorogenic TaqMan MGB probes and primers designed by Assay-on-Demand^TM^ gene expression products or predesigned qPCR assays from Integrated DNA Technologies, IDT. Mouse assay IDs were *Ccl2*: Mm00441242_m1, *Ccn2*: Mm01192933_g1, *Cdkn1a*: Mm00432448_m, *Cdkn2a*: Mm00494449_m1, *Col1a2*: Mm00483888_m1, *Fn1*: Mm01256744_m1, *Havcr1*: Mm00506686_m1, *Il1β:* Mm00434228_m1, *Il6*: Mm00446190_m1, *Il-18*: Mm00434226_m1, *Klotho:* Mm00502002_m1, *Lcn2*: Mm01324470_m1, *Nlrp3*: Mm00840904_m1, and *Serpine1:* Mm00435858_m1. Data were normalized to *Gapdh*: Mm99999915_g1. The mRNA copy numbers were calculated for each sample by the instrument software using the Ct value (“arithmetic fit point analysis for the lightcycler”). Results were expressed in n-fold, calculated relative to the control CCN2^+/+^ group after normalization against *Gapdh*.

### 4.7. Statistical Analysis

Kidneys from all groups were compared to control CCN2^+/+^, expressing results as a fold change over control values of 1, except for the PAS quantitation and the p21, which were expressed in arbitrary units, and the double immunofluorescence of gH2AX and Ki67, which was expressed in percentage. Results are expressed as a fold increase with respect to the control average as the mean ± standard error of the mean (SEM) of 4 to 10 animals per group. The Shapiro–Wilk test was used to evaluate sample normal distribution. When samples followed the Gaussian distribution, a one-way ANOVA followed by the corresponding post-hoc analyses was used. To compare non-parametric samples, a Kruskal–Wallis and a subsequent post-hoc analysis were performed. Graphics and statistical analysis were conducted using GraphPad Prism 9.5.1 (GraphPad Software, San Diego, CA, USA). Values of *p* < 0.05 were considered statistically significant.

## 5. Conclusions

CCN2 exerts pleiotropic actions due to its dual role as a matricellular protein and a growth factor and is involved in multiple relevant biological processes [102,103]. The present findings contribute to a better understanding of the CCN2 biology and reinforce the importance of its role as a driver of renal fibrosis and vascular rarefaction, mainly mediated by the activation of cellular senescence and favoring the AKI-to-CKD transition and renal damage progression.

## Data Availability

All data supporting the reported results are included in the manuscript or are available from the authors upon request.

## Abbreviations

The following abbreviations are used in this manuscript:

AKI	Acute Kidney Injury
BCL-xL	B-cell lymphoma-extra large
CCA	Cell cycle arrest
Ccl2	Chemokine (C-C motif) ligand 2
CCN2	Cellular communication network factor 2
Cdkn1a	Cyclin-dependent kinase inhibitor 1A (p21)
Cdkn2a	Cyclin-dependent kinase inhibitor 2A (p16)
CKD	Chronic Kidney Disease
Col1a2	Collagen type I alpha 2 chain
DDR	DNA Damage Response
ECM	Extracellular Matrix
FA	Folic acid
FAN	Folic-acid nephropathy
Fn1	Fibronectin 1
Havcr1	Hepatitis A Virus Cellular Receptor 1
HUVEC	Human Umbilical Vein Endothelial Cells
IL1-β	Interleukin 1 β
Il6	Interleukin 6
IRI	Ischemia-Reperfusion Injury
Ki-67	Marker of proliferation Ki-67
KIM-1	Kidney Injury Molecule-1
Lcn2	Lipocalin 2
NF-KB	Nuclear Factor kappa-light-chain-enhancer of activated B cells
NGAL	Neutrophil Gelatinase-Associated Lipocalin
NLRP3	NOD-like receptor protein 3
SASP	Senescence-Associated Secretory Phenotype
Serpine1	Serpin Family E Member 1 (also known as PAI-1)
TECs	Tubular Epithelial Cells
γH2AX	Phosphorylated Histone H2AX (marker of DNA damage)
